# Effects of Treatment with Probiotics on Cognitive Function and Regulatory Role of Cortisol and IL-1β in Adolescent Patients with Major Depressive Disorder

**DOI:** 10.3390/life13091829

**Published:** 2023-08-29

**Authors:** Shaoli Shi, Shuyou Zhang, Lingming Kong

**Affiliations:** 1 Psychiatry Department, The 5th People’s Hospital of Luoyang, Luoyang 471027, China; shishaoli123@126.com; 2 Intervention Center of Mental Crisis, No.904 Hospital, Changzhou 213003, China; 1598879814@qq.com

**Keywords:** adolescence, major depressive disorder, cognitive disorder, cortisol, IL-1β, probiotics

## Abstract

The aim of this study was to investigate the effects of probiotics on cognitive function and the regulation of cortisol and IL-1β in adolescents with depression. All 180 participants were randomly assigned to a study group (treated with probiotics combined with sertraline hydrochloride) and a control group (treated with sertraline hydrochloride). The repetitive Neuropsychological State Test (RBANS) and Hamilton Depression Scale (HAMD) were administered to MDD patients. The levels of serum cortisol and IL-1β were detected using an ELISA kit. Except for speech function, factors including immediate memory, visual span, attention function, delayed memory, and the RBANS in the study group were significantly higher than those in the control group. The levels of cortisol and interleukin-1β in the study group were significantly downregulated compared to those in the control group. Except for speech function, the cortisol level was negatively correlated with the RBANS total score and other factors in the study group. Interleukin-1β was also negatively correlated with the RBANS total score and each factor score. Cortisol and interleukin-1β were predictors of the RBANS total score, which explained 46.80% of the variance. Cortisol had significant predictive effects on attention function and delayed memory, and interleukin-1β had significant predictive effects on visual span and speech function. It could be concluded that probiotics could improve cognitive function in adolescents with depression by regulating cortisol and IL-1β levels.

## 1. Introduction

Major depressive disorder (MDD) is a common mental disorder among adolescents. An epidemiological survey in Zhengzhou showed that 20.03% of middle school students have obvious depressive symptoms, and 22.41% may have suspected depressive symptoms; the positive detection rate for depressive symptoms among high school students was significantly higher than that for middle school students. In Beijing, the prevalence rate of depressive disorder among students aged 6–16, which had an age effect, was 2.29% (234/10,215 cases). The prevalence rate of depressive disorder increased with age; the key turning point occurred at the age of 12, while the peak was reached at age 15 [1,2]. Previous studies have found that patients with MDD have impaired cognitive functions such as information-processing speed, attention or alertness, working memory, word learning, visual learning, reasoning and problem-solving, and social cognition [3]. After conventional antidepressant treatment, negative emotion and sleep quality could be significantly improved, but there were still obvious cognitive impairments [4,5]. Middle school students are at a crucial stage of development in academic performance. However, impaired cognitive function affects learning efficiency and correspondingly increases academic pressure; it may further induce or aggravate depressive symptoms. On the other hand, impaired cognitive function can affect treatment efficacy and increase the risk of relapse. This pathological process forms a vicious cycle whereby the depressive state of MDD patients is either retained or becomes more severe; therefore, intervention in and the rehabilitation of cognitive function remain an unsolved, worldwide problem in the clinical practice of treatment programs for MDD. 

The positive effects of probiotics on cognitive function have been preliminarily verified [6]. In one study, Fei found that probiotic supplementation could enhance cognitive functions and benefit sleep quality by increasing the abundance of intestinal flora in older adults with mild cognitive impairment [7]. First, Sanborn enrolled 200 English-speaking, healthy middle-aged and older adults (aged 55–75) recruited from the community using social media and local advertisements. Then, the participants were randomized to either the probiotic or placebo group using random number generation. The results of this randomized clinical trial indicated that Lactobacillus GG (LGG) probiotic supplementation could promote the remission of anxiety and depression and enhance the cognitive domains of executive functioning and processing speed [8]. The researchers argued that it was necessary to translate preclinical data into clinical data, where evidence is more limited. Recent studies, to some degree, have bridged the preclinical–clinical gap and suggest the role of infectious components in the pathogenesis of Alzheimer’s disease (AD); in cognitively impaired elderly patients with brain amyloidosis, there is a lower abundance of *E. rectale* and *B. fragilis* in the gut. In patients with AD, lactobacilli- and bifidobacteria-based probiotic supplementation improved cognitive, sensory, and emotional functions and general life quality [9]. In summary, in the present studies, probiotic supplementation was mainly utilized in improving cognitive function in patients with AD and MCI and healthy middle-aged and older adults, however, it may have potential applicable value in alleviating cognitive impairments of MDD patients. 

The acute symptoms of MDD are anhedonia, distress, insomnia, hopelessness, suicidal ideation and behavior, and non-suicidal self-injury (NSSI), not including cognitive impairment; therefore, therapeutic strategies, especially probiotic supplementation for cognitive impairment in MDD patients, are seldom explored. Another critical concern is that the mechanism of the improvement effect is still unclear. For example, present research results are sometimes contradictory. In one study, the kynurenine in the serum of MDD patients was lower than that of health volunteers. On the contrary, another double-blind study showed that the kynurenine of MDD patients was higher than that of healthy controls and that treatment with selective serotonin reuptake inhibitors (SSRI) combined with probiotic bacteria Lactobacillus plantarum 299v (LP299v) could lower the decreased KYN concentration and improve cognitive performance [6,10]. In recent years, the gut–microbiota–brain axis hypothesis of MDD has become an important field of pathological mechanisms for MDD. There is a two-pathway regulation between intestinal microbes and the central nervous system (CNS), and the disturbance of intestinal microflora can affect hippocampal neurogenesis and impair memory function in MDD patients; meanwhile, probiotics can regulate intestinal microecology and further improve cognitive function [11,12]. Gut microbiome disturbances have been widely implicated in MDD. One previous study suggested that Bacteroides species enriched in the gut microbiome from MDD patients differentially impact the susceptibility to depressive behaviors, and transplantation of a fecal microbiome from MDD patients into antibiotic-treated mice could induce anxiety and despair-like behavior and impair hippocampal neurogenesis [11]. In one prospective study, Zhang proved that the abundance of fecal Streptococcus was highly correlated with HAMD and HAMA scores. Patients with severe depression symptoms showed a higher abundance of Phascolarctobacterium and Akkermansia, while enrichment of Akkermansia, Coprococcus, and Streptococcus was observed with severe anxiety symptoms. Finally, the researchers argued that the fecal microbial metabolite indole-3-carboxyaldehyde proved useful in discriminating the severity of depression or anxiety symptoms in MDD patients [13]. Bruce-Keller in one review argued that communication pathways between gut microbiota and the CNS could include autonomic, neuroendocrine, enteric, and immune systems, with pathology resulting in disruption to neurotransmitter balance, increases in chronic inflammation, or exacerbated hypothalamic-pituitary-adrenal (HPA) axis activity [14]. It has been verified that inhibitory resource availability of the dorsal anterior cingulate cortex (dACC) enables successful learning and γ-aminobutyric acid (GABA, the brain’s major inhibitory neurotransmitter) levels, by potentially reflecting the recruitment of inhibitory systems during high cognitive load when trying to learn. Increased on the uncertainty condition, higher GABA levels during the null condition correlated with improved discrimination learning [15]. The stress response involves the activation of the HPA axis which may lead to the production of glucocorticoids (GCs). Being soluble in lipids, GCs easily cross the blood brain barrier and access GC receptors in the hippocampus, prefrontal cortex and amygdala, which are differentially involved in various cognitive functions of memory, emotion regulation, encoding of emotional memories, etc. [16]. Therefore, this study hypothesized that probiotics can affect brain function and furtherly improve cognitive performance in MDD patients through the gut-microbiota-brain (GMB) axis. Species of intestinal flora, which are abundant and diverse in the human body, may interact with each other, so intestinal flora disorder usually has a systematical feature, and it is difficult for a single flora species of probiotic supplementation to remodulate the intestinal flora balance. A new polypill combining Bifidobacterium, Lactobacillus, Enterococcus and Bacillus cereus into tablets (live) was employed in this study for the treatment of cognitive impairment in MDD patients with fewer clinical side effects. This polypill could supplement more than 98% of intestinal flora and efficiently restore the flora balance [17,18].

Cortisol and IL-1β are considered novel biomarkers of chronic stress, which trigger the impairment of the intestinal mucosal barrier function associated with MDD; animal models could also duplicate the aforementioned result. Previous studies have verified that probiotics could regulate the process of human inflammatory reactions and the release of stress hormones [19,20,21,22,23]. Cortisol acts as a pathway in the association between depressive symptoms and cognitive function; John found that direct effects of affective symptoms were shown across early to middle adulthood on cognitive function in midlife, including memory and information processing errors. Llorens concluded from one study that HPA axis hormone levels (plasma prolactin and cortisol levels and salivary cortisol) were associated with the severity of cognitive and inattention symptoms of patients with attention deficit and hyperactivity disorder and that childhood maltreatment and sex exert distinct moderating effects depending on the symptom type [24,25]. Increasing evidence supports an implication of immune system vulnerability and inflammatory processes on social cognition and emotion recognition, and illustrates the importance of interleukin-1β (IL-1β) in cognition processes dependent on the hippocampus. It also reinforces the fact that alpha-melanocyte-stimulating hormone (α-MSH) can reverse IL-1β effects on memory reconsolidation [26,27]. Therefore, we can hypothesize that cortisol and IL-1β are regulators between probiotics and the improvement of cognitive function in MDD patients. This study aimed to investigate the effect of probiotics on improving cognitive function in adolescents with MDD and the regulatory role of cortisol and IL-1β in the pharmacological mechanism.

## 2. Materials and Methods

### 2.1. Participants

This study was conducted in the 5th Affiliated Hospital in Science and Technology clinics, University of Henan, and all clinical data were collected from January 2019 to December 2021. A total of 160 adolescents with MDD, aged 17–19 years old, including 71 males and 79 females, were continuously enrolled by a convenient sampling method. Two psychiatrists diagnosed the patients according to the diagnostic criteria of MDD defined in the *Diagnostic and Statistical Manual of Mental Disorders*, 5th Edition (DSM-V) [28]. The inclusion criteria included ① senior high school students and ② all patients who were first diagnosed with MDD. The exclusion criteria included ① patients who came from divorced parents or whose parent(s) passed away; ② patients with a personal history of being left behind; ③ patients with severe medical disease or disability; ④ patients with a history of brain injury, poisoning, or asphyxia; and ⑤ patients who experienced significant life events in the previous six months, such as feeling lovelorn, experiencing an unanticipated accident, going through bereavement, etc.

Using a random number table, the experimenter randomly assigned patients to two rooms; consulting room 1 as the study group and consulting room 2 as the control group.

This study was approved by the Ethical Review Committee for Medical Research (NO.2019-2019-6-4), and written informed consent was obtained from all the participants.

### 2.2. Medical Intervention

The study group was assigned to receive treatment with probiotics combined with Sertraline, while the control group was treated with Sertraline only. The initial dose of Sertraline (Pfizer) for adolescent MDD patients is usually 50 mg/d and reaches a fixed dose of 100 mg per day after 4 to 7 days. All MDD patients have a dose range of 100 to 200 mg per day according to the body weight, gender, treatment response, side effect, depression severity, which may be appropriately adjusted according to the patient’s tolerance, body weight, gender, treatment response, side effect and depression severity. Two and seven patients in the study and control groups, respectively, reached the highest therapeutic dose of 200 mg per day; the therapeutic doses of the rest patients were 100 or 150 mg per day. The side effects, reported in this study including hypersomnia, hypodynamia and constipation, are in control after Sertraline dosage adjustment and behavioral intervention (diet, exercise, etc.). Probiotics (combined Bifidobacterium, Lactobacillus, Enterococcus and Bacillus cereus tablets, live, Hangzhou Yuanda Biology) were taken orally at 0.5 g at a time, twice daily. The treatment period of this study was 2 months.

### 2.3. Mental Assessment

#### 2.3.1. Repeatable Battery for the Assessment of Neuropsychological Status (RBANS)

The RBANS is utilized to evaluate cognitive performance in patients with MDD, schizophrenia, bipolar disorder, stroke, and Parkinson’s disease. The RBANS has good properties of reliability and validity and consists of 12 sub-tests (i.e., list learning, story memory, figure copy, line orientation, picture naming, semantic fluency, digit span, coding, list recall, list recognition, story recall, and figure recall), which are assigned into five factors of immediate memory, visuospatial abilities, language, attention, and delayed memory for a total scale score. The calculation method for the Randolph cortical–subcortical deviation score is as follows: [(visuospatial-construction + attention)/2] − [(language + delayed memory)/2], where scores > 0 indicate a “subcortical” pattern and scores < 0 indicate a “cortical” pattern of performance. The scores from the five domains contribute to an overall total RBANS score, and the administration time is approximately 30 min [29].

#### 2.3.2. Hamilton Depression Scale (HAMD)

The HAMD, consisting of 24 items, was employed to assess the severity of depressive symptoms in MDD patients. All items can be assigned into seven dimensions: anxiety/somatization, weight, cognitive disorder, diurnal variation, retardation, sleep disturbance, and hopelessness. The HAMD included 24 items; 10 items were scaled from 0 to 2, and the remaining 14 were scaled from 0 to 4. Items with 0–2 points were valued as none (0), mild-moderate (1), or severe (2), while items with 0–4 points were valued as none (0), mild (1), moderate (2), severe (3), or very severe (4); the higher the score, the more severe the degree of depression. The administration time was approximately 15 min [30].

The cognitive function and severity of depression in all MDD patients were anonymously rated before starting the medication, and the demographical variables of gender and age were also registered.

### 2.4. Cortisol and IL-1β Test

Participants abstained from a high-fat diet for 12 h and slept for 8 h with an overnight fast. Between 7:00 and 9:00 a.m., 5 mL of whole blood was collected in EDTA-containing anticoagulant tubes, which were then placed at room temperature for half an hour. The next step was to isolate the serum at a low temperature of 4 °C with 3000 r/min for 10 min, and then add the prepared species and standard product into the isolated serum to react for 30 min at 37 °C. After the plate was washed five times, the TMB chromogen solutions A and B were added for color display at 37 °C within 10 min. The last step was to add a termination solution and read the optical density (OD) value within 5 min. Cortisol and IL-1β were detected using an enzyme-linked immunosorbent assay (ELISA kit, purchased from Shanghai Mingfeng Biological Co., Ltd., Shanghai, China, Art. No. M-10718, M-10083). A 10*96-well plate was precoated with anti-cortisol/IL-1β IgG. Samples and the cortisol/IL-1β-horseradish peroxidase (HRP) conjugate were added to the wells, where any cortisol/IL-1β in the sample could compete with the added cortisol/IL-1β-HRP for antibody binding. The wells were washed to remove unbound material after incubation, and then a tetramethylbenzidine (TMB) substrate was added, which was catalyzed by HRP to produce blue coloration. The reaction was terminated by adding a stop solution, which stopped the color development and produced a color change from blue to yellow. The intensity of the signal was inversely proportional to the amount of cortisol/IL-1β in the sample, and the intensity was measured at 450 nm. All the procedures were carried out according to the instruction manual provided by the manufacturer.

### 2.5. Statistical Analysis

SPSS21.0 was used for data management and analysis, χ^2^ tests were used for discrete variables, an independent sample *t*-test was used for continuous variables, and Pearson’s correlation analysis and regression analysis were conducted for data processing [31]. *p* < 0.05 was considered statistically significant.

## 3. Results

### 3.1. Between-Group Comparison of Demographic and Clinical Variables

There were no significant differences in gender, age, and HAMD scores between the two groups (*p* > 0.05) (see Table 1).

### 3.2. Comparison of Cognitive Functions between the Study Group and Control Group

An independent sample *t*-test showed that the factor scores of immediate memory, visuospatial abilities, attention, and delayed memory, except for language, and the total RBANS score were significantly higher in the study group than those in the control group (*p* < 0.05 or 0.01) (see Table 2).

### 3.3. Comparison of Cortisol and IL-1β between the Study Group and Control Group

An independent sample *t*-test revealed that the levels of cortisol and interleukin-1β in the study group were significantly lower than those in the control group (*p* < 0.05 or 0.01) (see Table 3).

### 3.4. Correlation Analysis of Cognitive Function and Cortisol and IL-1β in the Study Group

Pearson’s correlation analysis was performed for cognitive function and cortisol and IL-1β in MDD patients. In addition to the factor of language, the cortisol level in the study group was negatively correlated with the total RBANS score and other factor scores (*p* < 0.01); IL-1β was negatively correlated with the total score and factor scores of RBANS (*p* < 0.05 or 0.01) (see Table 4).

### 3.5. Regression Analysis of Associated Factors of Cognitive Function in the Study Group

A multiple regression analysis was employed for data analysis with factor scores and a total score of RBANS as dependent variables and cortisol and IL-1β as independent variables. The results indicated that cortisol and IL-1β were predictors of RBANS total scores (*p* = 0.000), which accounted for 46.80% of the variance. Cortisol significantly predicted attention and delayed memory, and IL-1β significantly predicted visuospatial abilities and language (*p* < 0.05 or 0.01) (see Table 5).

## 4. Discussion

Development and health maintenance have a transitional meaning in the milestones of each adolescent’s life, and adolescents are commonly susceptible to various kinds of stress. Yang et al., in one survey, found that adolescents tended to experience a stress response to the epidemic of COVID-19, which was associated with their depressive symptoms. Fu et al., in another study, argued that traditional Confucianism and examination culture resulted in Chinese families having high expectations for the academic performance of teenagers. The previous study showed that parent-child communication and interaction style could affect teenagers’ perceptions of academic pressure, which, combined with too many learning tasks, led to inadequate exercise time and weight gain; being overweight may furtherly augment a teenager’s susceptibility to a stress response. It has been verified that the academic pressure of junior high school students in China is associated with school burnout and eventually induced depressive symptoms [32,33,34]. The conventional therapeutic response to cognitive impairment, which is usually considered the core symptom of MDD, is poor. In present clinical practice, improving treatment efficacy and prognosis for cognitive impairment is an urgent task.

This study found that the immediate memory, visuospatial abilities, attention, delayed memory, and total RBANS score were higher, and the levels of cortisol and IL-1β were lower in the study group than those in the control group. Cortisol and IL-1β were negatively correlated with the total score and factor scores of RBANS. Multiple regression analysis showed that cortisol and interleukin-1β were predictors of the total RBANS score, accounting for 46.80% of its variance. Cortisol is a significant predictor of attentional and delayed memory, and IL-1β had a predictive effect on visuospatial abilities and language. These results indicate that probiotics can improve cognitive function in adolescents with depression by regulating the levels of cortisol and IL-1β.

In recent years, the GMB axis theory, which poses a continuous bidirectional model of regulation between the CNS and the gastrointestinal tract (GT), has become a hot field regarding pathological mechanisms for MDD. Changes in intestinal microbial composition can increase the permeability of the intestinal barrier and activate inflammation and the immune response of the whole body. This aforementioned process, which further regulates the release of monoamine neurotransmitters by alerting the activity of the HPA axis and abundance of the brain-derived neurotrophic factor (BDNF), eventually leads to MDD [35,36,37]. Cortisol is a kind of steroid hormone produced by the adrenal gland for stress response, while IL-1β is a cytokine that activates and regulates the function of immune cells and participates in the regulation of human inflammatory response. Both cortisol and IL-1β are sensitive to environmental stress, significantly affecting an individual’s ability to maintain normal physiological function and adapt to the environment. Heavy learning tasks, high academic competition pressure, and common sleep debt increase the levels of cortisol and IL-1β. Previous studies have confirmed that cortisol can interact with testosterone to reduce the volume of the hippocampus and impair episodic memory; meanwhile, interleukin-1β, known as the “cytokine storm”, can induce secretion of other inflammatory cytokines and result in a neuron reduction in hippocampal pyramidal cells and the weakening of neural regeneration in the dentate gyrus. At the same time, a high level of IL-1β can decrease BDNF and trigger the atrophy and, ultimately, death of hippocampal neurons [38,39,40]. In addition, other studies have found that the volume of the prefrontal lobe in individuals who experience chronic and stable stress is smaller than that in the controls. Meanwhile, the decrease in hippocampus volume caused by chronic stress, consistent with the upregulation of cortisol and IL-1β, is considered one of the neural bases of MDD [41,42,43,44]. It can further affect cognitive functions of reasoning, problem-solving, planning and strategy, analysis, behavioral decision making and inhibition, memory, etc., in patients with MDD.

Long-term chronic stress featured with high levels of cortisol and IL-1β can cause disorders of intestinal flora composition, one recent study showed that the depression mice model established by Chronic Unpredictable Mild Stress (CUMS) had obvious intestinal flora disorder and Cinnamon Oil Solid Self-Microemulsifying Drug Delivery System (CO-S-SME) changed the intestinal flora composition by decreasing the ratio of Firmicutes to Bacteroidetes, reducing relative abundances of Lactobacillus, modulating Alpha diversity and Beta diversity [45]. Appropriate supplementation of probiotics can promote the restoration of balance and functional improvements in the intestinal flora. Based on the bidirectional model of regulation between the CNS and GT, probiotics, which are beneficial for the maturity of intestinal epithelial cells and maintenance of the intestinal barrier function, can induce changes in the expression and levels in the circulation of pro-inflammatory and anti-inflammatory cytokines, directly affecting brain function. Otherwise, probiotics may limit the invasion of endotoxins by changing intestinal permeability and abating inflammatory responses, inhibiting the hyperactivity of the HPA axis [46,47]. In a word, a decrease in cortisol and IL-1β levels can ultimately improve the cognitive function of adolescents with MDD.

One limitation of the present study is that the small sample size may have made the results susceptible to type II errors; therefore, any data must be cautiously interpreted with respect to other MDD patient groups. Another limitation is that life quality and psychosocial function were not assessed. It is necessary to further verify this study’s results in a large sample and conduct a longitudinal study to observe the life quality, psychosocial function, rehabilitation rate, and academic performance of MDD patients in future research. Based on the findings in this study, there may be hope for efficiently improving the cognitive function of MDD patients in clinical settings.

## 5. Conclusions

The present study verifies that probiotics improve cognitive function in adolescents with MDD by regulating the levels of cortisol and IL-1β through a mechanism involving the microbial–gut–brain axis. This study indicated that probiotics combined with antidepressants should be emphasized in clinical practice to improve the prognosis of MDD.

## Figures and Tables

**Table 1:** Between-group comparison of demographic and clinical characteristics (%/*x* ± SD).

Variables	Study Group (N = 80)	Control Group (N = 80)	*χ* ^2^ */t*	*p*
Gender				
Female	34	37	0.98	0.223
Male	46	43		
Age	17.86 ± 0.67	17.68 ± 0.79	0.26	0.794
HAMD score	31.08 ± 5.96	30.84 ± 5.52	1.62	0.108
RBANS score	281.43 ± 37.90	271.18 ± 41.35	1.63	0.104

**Table 2:** Comparison of cognitive functions between the study group and control group (*x* ± SD).

Factors	Study Group (N = 80)	Control Group (N = 80)	*t*	*p*
Immediate memory	65.93 ± 17.26	60.16 ± 15.79	2.20	0.029
Visuospatial abilities	90.69 ± 21.30	83.70 ± 19.62	2.16	0.032
Language	79.89 ± 11.12	82.18 ± 11.48	−1.28	0.202
Attention	87.44 ± 11.00	81.70 ± 15.57	2.69	0.008
Delayed memory	64.61 ± 19.75	56.73 ± 12.94	2.99	0.003
Total score of RBANS	388.55 ± 46.72	364.46 ± 44.43	3.34	0.001

**Table 3:** Comparison of cortisol and interleukin-1β between the study group and control group (*x* ± SD).

Indices	Study Group	Control Group	*t*	*p*
Cortisol	234.22 ± 32.01	633.44 ± 70.23	−5.23	0.000
IL-1β	86.82 ± 25.61	130.11 ± 55.80	−2.45	0.015

**Table 4:** Correlation analysis of cognitive function and cortisol and IL-1β in the study group (*r*).

Indices	Immediate Memory	Visuospatial Abilities	Language	Attention	Delayed Memory	Total Score of RBANS
Cortisol	−0.293 **	−0.378 **	−0.106	−0.376 **	−0.519 **	−0.614 **
IL-1β	−0.257 *	−0.451 **	−0.311 **	−0.380 **	−0.240 *	−0.565 **

Note: * is *p* < 0.05, ** is *p* < 0.01.

**Table 5:** Regression analysis of associated factors of cognitive function in the study group.

Dependent Variable	Independent Variable	Regression Coefficient	Standard Error	*t*	*p*	*R* ^2^
Immediate memory	Cortisol	−0.029	0.016	−1.78	0.080	0.103
	IL-1β	−0.100	0.084	−1.20	0.235	
Visuospatial abilities	Cortisol	−0.033	0.018	−1.81	0.075	0.236
	IL-1β	−0.291	0.095	−3.05	0.003	
Language	Cortisol	0.005	0.010	0.51	0.615	0.100
	IL-1β	−0.149	0.054	−2.76	0.007	
Attention	Cortisol	−0.021	0.010	−2.13	0.037	0.192
	IL-1β	−0.110	0.051	−2.18	0.032	
Delayed memory	Cortisol	−0.079	0.017	−4.73	0.000	0.269
	IL-1β	0.016	0.086	0.18	0.857	
Total score of RBANS	Cortisol	−0.157	0.034	−4.64	0.000	0.468
	IL-1β	−0.634	0.174	−3.64	0.000	

## Data Availability

The data and trial protocol can by requested from the corresponding author.

## Abbreviations

Major depressive disorder, MDD; interleukin-1β, IL-1β; Repeatable Battery for the Assessment of Neuropsychological Status, RBANS; Hamilton Depression Scale, HAMD; central nervous system; CNS; gastrointestinal tract, GT; hypothalamic-pituitary-adrenal axis, HPA axis; brain-derived neurotrophic factor, BDNF.
